# Shear Behavior of GMTC/BPC-GCL Interface Under Dry and Hydrated Conditions with Varying Polymer Content

**DOI:** 10.3390/polym18121423

**Published:** 2026-06-07

**Authors:** Juan Hou, Zhenyi Shi, Xuelei Xie

**Affiliations:** 1 School of Mechanics and Engineering Science, Shanghai University, Shanghai 200444, China; shizhenyi@shu.edu.cn (Z.S.); xiexuelei@shu.edu.cn (X.X.); 2 School of Engineering, University of Virginia, Charlottesville, VA 22904, USA; 3 School of Civil Engineering and Water Resources, Qinghai University, Xining 810016, China

**Keywords:** geosynthetic clay liner, polymer-modified bentonite, coextruded textured geomembrane, interface shear behavior, hydration, direct shear tests

## Abstract

Polymeric geosynthetics serve as fundamental components of engineered composite liners in waste containment facilities. The interface shear behavior between a coextruded textured geomembrane (GMTC) and a bentonite–polymer composite geosynthetic clay liner (BPC-GCL) was investigated under both dry and hydrated conditions, with varying polymer content (0%, 3.5%, and 5.5%), using large-scale direct shear tests. Hydration of BPC-GCL was found to significantly reduce GMTC/BPC-GCL interface shear strengths, with the magnitude of reduction increasing with normal stress. For the BPC-GCL with 3.5% polymer content, the peak strength at 400 kPa decreased by 36% from 272 kPa (dry) to 175 kPa (hydrated), which was attributed to bentonite softening and reduced frictional resistance. Polymer content non-linearly influenced shear behavior. At 400 kPa, the 3.5% BPC-GCL exhibited an 18% higher peak strength than the conventional GCL, while the 5.5% BPC-GCL showed a 9% reduction compared to the 3.5% specimen, attributed to internal structural damage and interfacial lubrication. Visual post-shear inspections revealed that dry conditions promoted interfacial friction-dominated failure, while hydration induced significant internal BPC-GCL damage, including fiber break and bentonite extrusion. The failure mode shifted with polymer content, and conventional GCL failed through internal bentonite deformation, while BPC-GCL exhibited a composite mechanism combining internal reinforcement and interfacial friction, with the 3.5% BPC-GCL demonstrating a more favorable composite effect than the 5.5% BPC-GCL. The study underscored the critical roles of hydration conditions and polymer modification in governing the shear mechanisms and strength at the GMTC/BPC-GCL interface.

## 1. Introduction

Polymeric geosynthetic materials provide advanced and resilient anti-seepage solutions for geotechnical and environmental infrastructure. They have driven the technical evolution of high-performance containment systems and waste isolation technologies, ensuring the reliable long-term isolation of hazardous pollutants [1,2]. This progress is primarily attributed to the unique macromolecular structures and superior physicochemical properties of polymers, such as exceptional chemical resistance and low permeability [3,4].

Composite liner systems, consisting of a high-density polyethylene (HDPE) geomembrane (GM) over a geosynthetic clay liner (GCL) or a bentonite–polymer composite GCL (BPC-GCL), are essential barriers in modern waste containment facilities to prevent pollutant migration [2,5,6]. The integrity and long-term stability of these systems depend largely on the shear strength at the GM-GCL or GM/BPC-GCL interface, which often forms the critical slip surface [6,7,8,9]. The interfacial shear behavior is governed by a complex interplay of factors, including the surface texture of the GM (e.g., coextruded, impinged, embossed), the characteristics of the GCL (such as geotextile type, granular or powdered bentonite, and the presence or absence of polymer), the hydration state of the bentonite, the applied normal stress, and the potential for extrusion of bentonite or other constituents from the GCL or BPC-GCL [8,10,11,12,13,14,15,16,17,18,19,20]. Among these factors, hydration plays a particularly important role. Hydration has been shown to significantly reduce the shear strength of the GM/GCL interface, with the interfacial shear response strongly influenced by the hydration of the bentonite within the GCL [11,15]. Under hydrated conditions, bentonite is more susceptible to extrusion than under dry conditions, and this extrusion has been found to markedly affect the interface shear strength [12,13,21].

Few studies have reported that the interfacial shear strength of BPC-GCLs is generally lower than that of conventional GCLs [18,22,23]. This reduction is primarily attributed to the leaching and subsequent migration of polymer toward the interface during hydration, where it forms a lubricating hydrogel layer that creates a weak shear plane [22,23,24,25]. This effect becomes more pronounced with increasing polymer content and higher applied normal stress [18,24,25]. However, studies that systematically compare the interface shear behavior of BPC-GCLs under both dry and hydrated conditions, and further examine the influence of varying polymer content under hydrated conditions, remain limited in the literature [17,18,22,23,24,25,26,27,28].

This study presents a comprehensive experimental investigation of the interface shear behavior between a widely used coextruded textured geomembrane (GMTC) and BPC-GCL under both dry and hydrated conditions with varying polymer content. For comparison, the interface shear behavior of the GMTC-GCL system was also evaluated. Large-scale direct shear tests were conducted over a range of normal stresses, aiming to quantify the peak and large-displacement strengths, analyze the deformation behavior, and identify the underlying failure mechanisms. The findings are intended to provide a scientific foundation for the appropriate polymer content, interface stability design, and long-term safety evaluation of polymeric composite liners in variable hydration environments.

## 2. Materials and Methods

### 2.1. Geosynthetics

**Figure 1:**
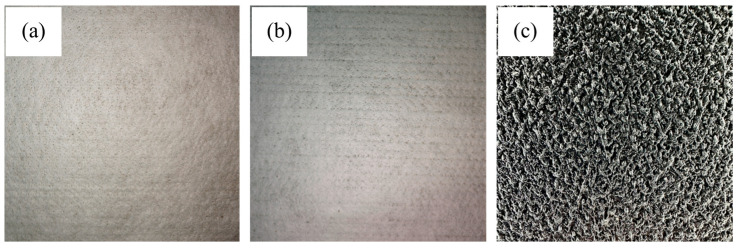
Photographs of materials used in the large-scale shear tests: (**a**) GCL; (**b**) BPC-GCL; (**c**) coextruded textured geomembrane (GMTC).

The GCL and BPC-GCL employed in the shear tests comprised granular bentonite sandwiched between a 200 g/m^2^ nonwoven geotextile and 110 g/m^2^ woven geotextile bonded by needlepunching (Figure 1a,b). Both geotextiles were polypropylene. The primary distinction lies in the incorporation of proprietary polymers within the bentonite core of the BPC-GCL. The polymer has a backbone structure and molecular weight characteristics typical of water-soluble macromolecules. The polymer contents of the BPC-GCL were 3.5% and 5.5% in this study, which represent the mass percentage of the polymer additive determined relative to the dry weight of the bentonite. The properties of the GCL and BPC-GCL are summarized in Table 1.

The geomembrane used in this study was a coextruded textured high-density polyethylene (HDPE) sheet (GMTC) with a nominal thickness of 2.0 mm. A photograph of the surface of the geomembrane is shown in Figure 1c, and the properties of the geomembrane are summarized in Table 2.

### 2.2. Large-Scale Direct Shear Apparatus

**Figure 2:**
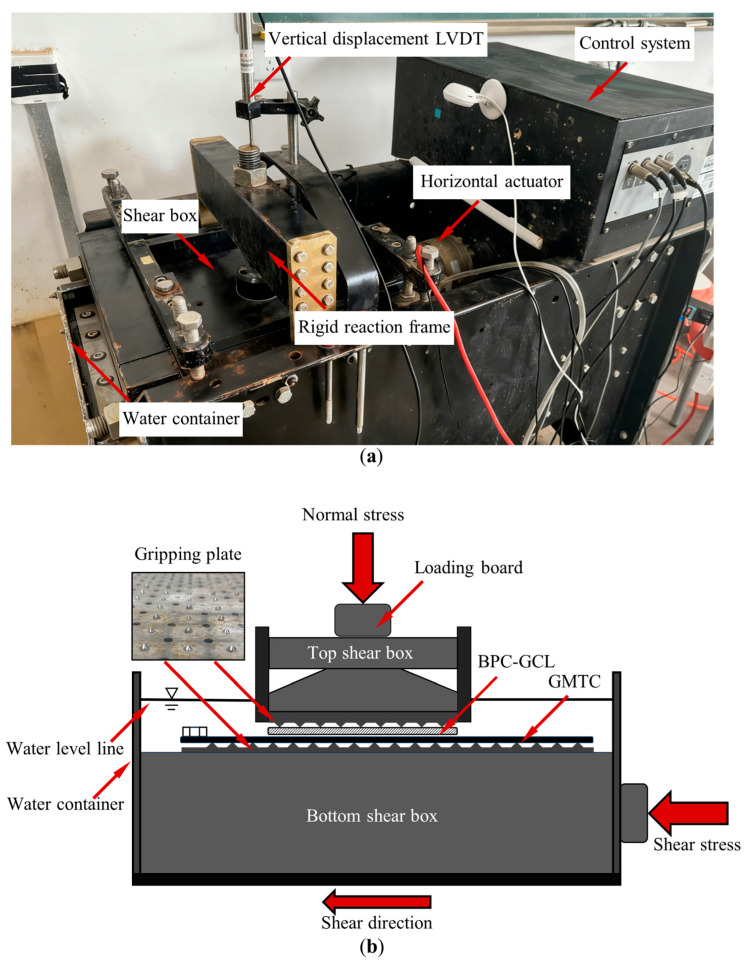
Large-scale direct shear apparatus used in texting program: (**a**) photograph and (**b**) schematic diagram.

The experiment employed an HM-5780 large-scale direct shear apparatus (Humboldt Mfg. Co., Elgin, IL, USA), as depicted in Figure 2. The maximum horizontal shear displacement of this direct shear apparatus is 100 mm, the maximum initial normal pressure is 45 kN, and the maximum shear rate is 10 mm/min. The upper shear box was 305 mm × 305 mm × 102 mm, and the lower shear box was 405 mm × 305 mm × 100 mm. Testing was conducted following Procedure B in ASTM D6243 [29,30]. The normal stress is applied through the loading head attached to a 30 mm thick steel plate (305 mm × 305 mm), and the loading head applies the normal stress to the interface through the gripping plate attached to the steel plate and in contact with the GCL or BPC-GCL surface. The lower box uses a similar steel gripping plate (Figure 2b). The horizontal actuator acts on the lower box, while the upper box is fixed. The shear stress and normal stress were recorded by a stress gauge, and the normal and vertical displacements at the GMTC-GCL or BPC-GCL interface were recorded by a linear variable displacement sensor (LVDTs). During the testing process, a personal computer equipped with a data acquisition card was used for data collection.

### 2.3. Test Procedures

**Figure 3:**
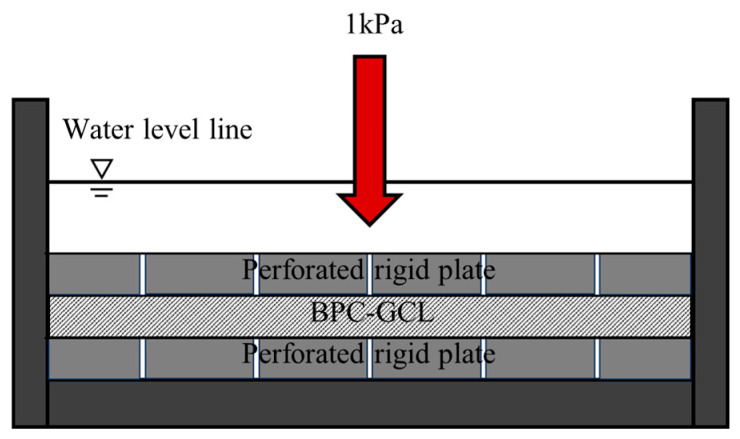
GCL or BPC-GCL hydration schematic diagram.

The three major steps of the direct shear tests conducted in this study are as follows:

(1) Preparation of test specimens: The GMTC and GCL or BPC-GCL specimens were taken from single rolls of the product and cut with the longitudinal axes parallel to the shear direction. The sizes of GMTC and GCL or BPC-GCL specimens were 305 mm × 405 mm and 305 mm × 305 mm, respectively. The GMTC specimens were 100 mm longer than the GCL or BPC-GCL specimens in the shear direction to ensure the shear area was maintained at 305 mm × 305 mm during shearing.
(2) Hydration of GCL or BPC-GCL specimens: The two-stage accelerated procedure proposed by Fox and Stark [6,31] was applied to hydrate GCL or BPC-GCL specimens in Figure 3. In the first stage, pre-cut GCL or BPC-GCL specimens were placed at the base of a hydration tank to ensure uniform vertical loading. A porous rigid plate was then positioned on top and bottom of the GCL or BPC-GCL, and deionized water (DIW) was added until the plate’s surface was submerged. Next, a vertical load of 1 kPa was applied, and the specimens were left undisturbed for 48 h to ensure complete hydration. In the second stage of hydration, GCL or BPC-GCL specimens were placed against the upper plate, and the GMTC specimens were affixed to the lower plate. The nonwoven side of the GCL or BPC-GCL was in contact with the GMTC, representing the orientation commonly used in practice [8,9,32]. The woven side was in contact with the steel gripping plate on the upper plate. Subsequently, a waterproof barrier was installed, and DIW was added to fully submerge the shear interface. Following the rapid consolidation test protocol, the normal stress was incrementally increased until reaching the target normal stress for the shear test. After a 24 h preloading period, vertical displacement tended to stabilize. The final water content of consolidated GCL or BPC-GCL is highly dependent on the applied normal stress level; this hydration protocol ensures the specimens reach an equilibrium state consistent with published baseline data [6,18,33,34].
(3) Shear tests and data recording: The shear process was initiated following the adjustment of parameters, including the shear displacement and shear rate [35]. Normal stresses of 100 kPa, 200 kPa, and 400 kPa were applied, with a final shear displacement set at 50 mm. A displacement of 50 mm was sufficient to illustrate how the post-peak loss in strength varied with hydration conditions and polymer content according to ASTM D6243 [36]. Hydrated specimens were sheared at a reduced rate of 0.1 mm/min to ensure fully drained conditions and prevent the generation of excess pore water pressures, whereas dry specimens were sheared at a rate of 1.0 mm/min [16,22]. While this testing protocol introduces a rate difference between the two states, a previous study by Feng [28] established that the interface shear strength of GMB/GCL systems under dry conditions is independent of the shear rate (0.1, 1, 10, 100 mm/min). Therefore, the significant reduction in shear strength observed in the hydrated specimens is fundamentally driven by the hydration phase transition of the bentonite and polymer, rather than the variation in shearing rates. Previous studies have shown that polymer content has a negligible effect on interfacial shear strength under dry conditions [18,24,27]. The BPC-GCL with 3.5% polymer content was selected as the representative baseline for dry testing. Details of the test program and corresponding test parameters are listed in Table 3. To ensure data reliability and address experimental variability, a selected representative case (3.5% BPC-GCL under hydrated conditions) was tested using two replicate specimens at 400 kPa, yielding peak shear strengths of 172 kPa and 179 kPa. The variations in the measured peak shear strengths among the replicates were within ±4.1%. In the subsequent sections, the average values obtained from replicate tests for this case are consistently adopted, and the corresponding error bars are explicitly presented in the relevant figures. However, to maintain consistency throughout the manuscript, a single representative specimen was selected for the photographic illustrations.

## 3. Results

### 3.1. Shear Stress–Shear Displacement Relationships

**Figure 4:**
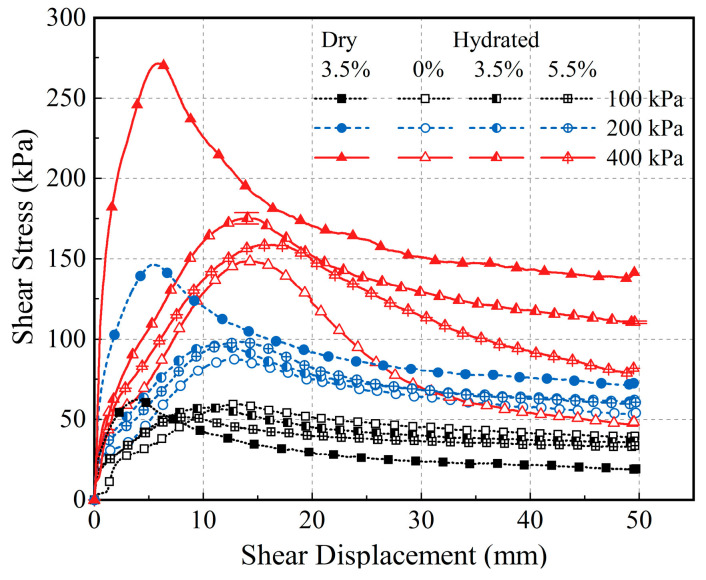
Shear stress–shear displacement relationship of the GMTC/BPC-GCL interfaces with varying polymer content under dry and hydrated conditions.

Figure 4 shows the shear stress–shear displacement relationship of the GMTC/BPC-GCL interfaces with varying polymer content under dry and hydrated conditions at 100 kPa, 200 kPa and 400 kPa.

Interfaces generally exhibit higher peak and large-displacement shear strengths under dry conditions. At a polymer content of 3.5%, the peak shear strengths under dry conditions are 62 kPa, 147 kPa, and 272 kPa at normal stresses of 100 kPa, 200 kPa, and 400 kPa, respectively. However, under hydrated conditions, the corresponding values are 57 kPa, 97 kPa, and 175 kPa, representing reductions of 8%, 34%, and 36%, respectively, compared to the dry conditions. Similarly, the large-displacement shear strengths under dry conditions are 19 kPa, 73 kPa, and 142 kPa, while under hydrated conditions they are 37 kPa, 62 kPa, and 111 kPa, showing changes of 94%, −15%, and −22%, respectively.

The influence of polymer content on shear strength under high normal stress (400 kPa) is much greater than that under low (100 kPa) and intermediate normal stresses (200 kPa) under hydration conditions. Comparison of these curves indicates relatively consistent peak and large-displacement shear strengths among the three polymer contents at low and intermediate normal stresses (100 kPa, 200 kPa). At 0% polymer content (conventional GCL), the peak shear strengths at low (100 kPa) and intermediate normal stresses (200 kPa) are 59 kPa and 87 kPa, respectively. At 3.5% polymer content, the corresponding values are 57 kPa and 97 kPa, while at 5.5%, they are 51 kPa and 98 kPa. Similarly, the large-displacement shear strengths at 0% polymer content are 39 kPa and 54 kPa under low (100 kPa) and intermediate normal stresses (200 kPa), respectively. At 3.5% polymer content, these values are 37 kPa and 62 kPa, and at 5.5%, these values are 33 kPa and 59 kPa. In contrast, more pronounced variations in peak shear strength are observed at high normal stress (400 kPa). Under a normal stress of 400 kPa, the peak shear strengths were 148 kPa, 175 kPa, and 159 kPa for polymer content of 0% (conventional GCL), 3.5%, and 5.5%, respectively. The peak shear strength at 3.5% polymer content increases by approximately 18% compared to that at 0% polymer content (conventional GCL). When the polymer content increases to 5.5%, the peak shear strength decreases by about 9% relative to the 3.5% composition. Similarly, under a normal stress of 400 kPa, the large-displacement shear strengths were 49 kPa, 111 kPa, and 82 kPa for polymer content of 0%, 3.5%, and 5.5%, respectively. The large-displacement shear strength at 3.5% polymer content was enhanced by approximately 128% compared to that of 0% polymer content (conventional GCL). However, when the polymer content was increased to 5.5%, the large-displacement shear strength was reduced by about 26% relative to the 3.5% polymer composition.

**Figure 5:**
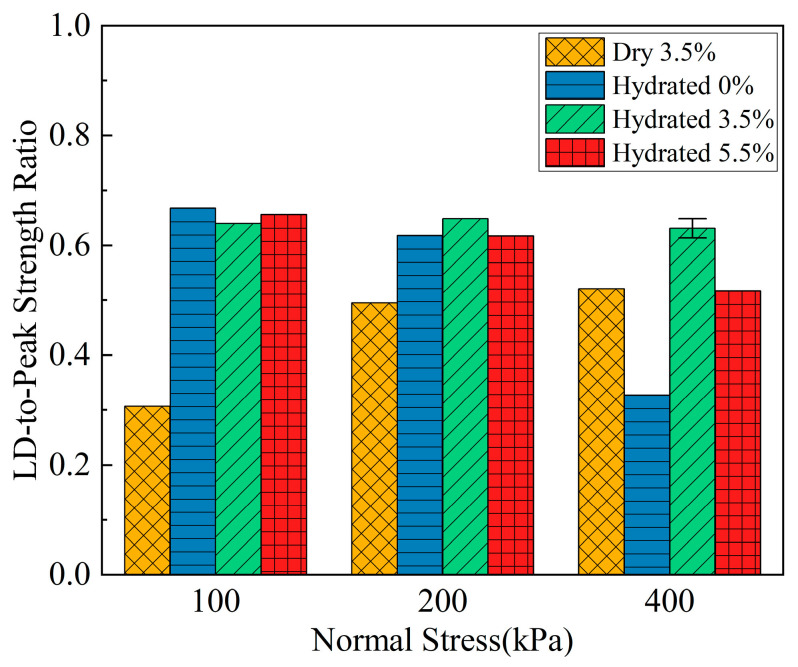
Large displacement (LD) to peak strength ratio of the GMTC/BPC-GCL interfaces with varying polymer content under dry and hydrated conditions.

Figure 5 shows the large displacement (LD) to peak strength ratio of the GMTC/BPC-GCL interfaces with varying polymer content under dry and hydrated conditions at 100 kPa, 200 kPa and 400 kPa. The peak shear strength is defined as the maximum shear stress on each shear-displacement curve, while the large-displacement strength is defined as the shear stress at 50 mm displacement [6,18]. The ratio of LD-to-peak strength is defined as the ratio of the large-displacement strength to the peak shear strength. A comparison of the ratios of LD-to-peak strength for the 3.5% polymer content BPC-GCL under dry and hydrated conditions revealed that the ratio of LD-to-peak strength under dry conditions was significantly lower than under hydrated conditions. However, this difference diminished with the increasing normal stress. When comparing the ratio of LD-to-peak strength corresponding to varying polymer content, it was revealed that the effect of polymer content was not substantial under low normal stresses (100 kPa and 200 kPa). In contrast, under a high normal stress of 400 kPa, the BPC-GCL with 3.5% polymer content exhibited the highest ratio of LD-to-peak strength among the three compositions. The conventional GCL demonstrated a significantly lower ratio of LD-to-peak strength compared to the BPC-GCLs with 3.5% and 5.5% polymer content. The variation in the ratio of LD-to-peak strength with normal stress was also examined. For the 3.5% BPC-GCL under dry conditions, the ratio of LD-to-peak strength increased with increasing normal stress. Under hydrated conditions, the ratio of LD-to-peak strength of the 3.5% BPC-GCL was less influenced by the normal stress, whereas the ratios for both the conventional GCL (0% polymer) and the 5.5% BPC-GCL decreased with increasing normal stress. Noticeably, at 400 kPa, the ratio of LD-to-peak strength of the conventional GCL decreased substantially compared to its values at lower normal stresses (100 kPa and 200 kPa), which was attributed to severe internal damage occurring at the GMTC/conventional GCL interface under this high stress level. The mechanisms responsible for these variations in shear curves and strength parameters are discussed in Section 4 in conjunction with photographic evidence.

### 3.2. Shear Strength Envelopes

**Figure 6:**
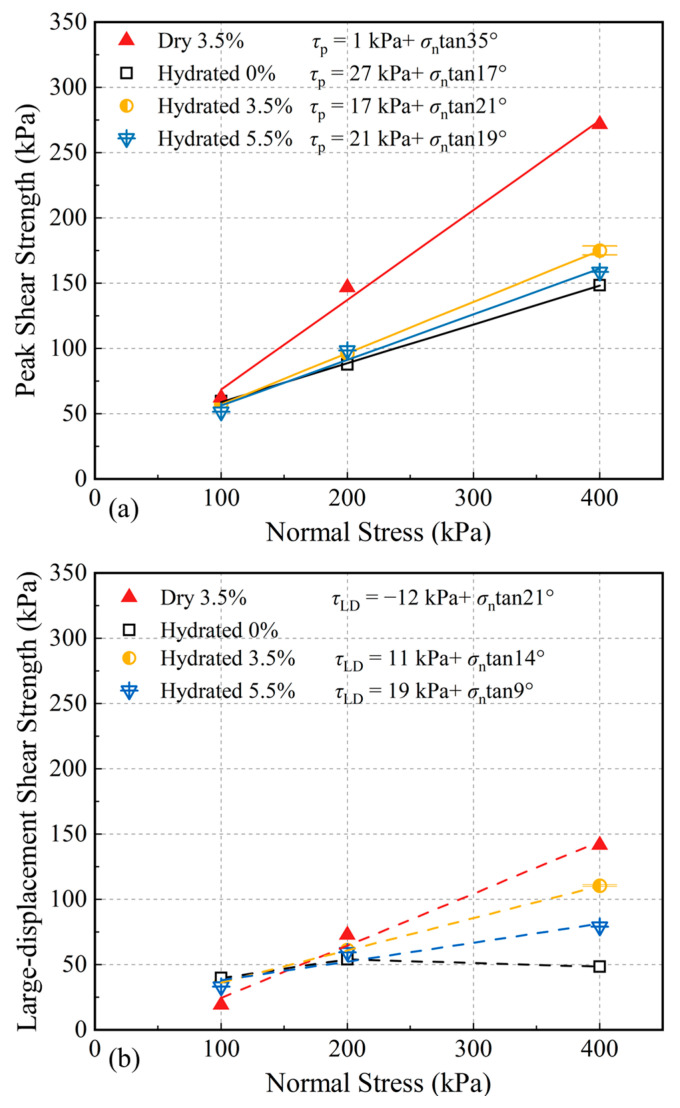
Peak and large-displacement interface strength envelopes for the GMTC/BPC-GCL interfaces with varying polymer content under drying and hydration conditions: (**a**) peak interface strengths and (**b**) large-displacement interface strength.

Peak and large-displacement interface strength envelopes for the GMTC/BPC-GCL interfaces with varying polymer content under drying and hydration conditions at 100 kPa, 200 kPa and 400 kPa are shown in Figure 6. The relationship between the shear strength and normal stress can be represented by the Mohr-Coulomb criterion:

(1) $$\tau_{p}=a_{p}+\sigma_{n}\tan\delta_{p}$$

(2) $$\tau_{\mathrm{LD}}=a_{\mathrm{LD}}+\sigma_{n}\tan\delta_{\mathrm{LD}}$$

where *τ*_p_ and *τ*_LD_ are the peak and large-displacement interface shear strength, *σ*_n_ is the normal stress, *a*_p_ and *a*_LD_ are the peak and large-displacement interface adhesion forces, *δ*_p_ and *δ*_LD_ are the peak and large-displacement interface friction angles. For most interfaces, the envelope of peak and large-displacement interface strength was generally approximately linear. Both Equations (1) and (2) demonstrated an excellent linear fit, with *R*^2^ values above 0.97. Under hydrated conditions at 400 kPa normal stress, the conventional GCL (0% polymer) sustained severe internal damage, and its large-displacement strength envelope exhibited nonlinearity (Figure 6b).

Comparison between dry and hydrated conditions indicates that both the peak and large-displacement shear strengths under dry conditions are generally higher than those under hydrated conditions, particularly under high normal stress. While the interface adhesion force under hydrated conditions (both at peak and large displacement) is higher than under dry conditions, the corresponding interface friction angles are lower. For the BPC-GCL interface with 3.5% polymer content, the peak interface friction angle measured under dry conditions was 35°, which decreased to 21° under hydrated conditions, representing a reduction of 14° (Figure 6a). Similarly, the large-displacement interface friction angle was 21° under dry conditions and decreased to 14° under hydration, also corresponding to a reduction of 7° (Figure 6b).

A comparative analysis of varying polymer content under hydrated conditions reveals a similar overall trend for both the peak and large-displacement interfacial shear strengths, with the 3.5% polymer content yielding the highest strengths, followed by the 5.5% polymer content and then the 0% polymer content (conventional GCL). As the polymer content increases, these strengths initially increase and subsequently decrease. As illustrated in Figure 6a, the peak interface adhesion force first decreases and then increases with increasing polymer content, while the peak interface friction angle exhibits an opposite trend, increasing initially and then decreasing. When the polymer content increased from 0% to 3.5%, the peak interface adhesion force decreased from 27 kPa to 17 kPa, while the peak interface friction angle increased from 17° to 21°. As the polymer content further increased from 3.5% to 5.5%, the interface adhesion force increased from 17 kPa to 21 kPa, and the peak interface friction angle decreased from 21° to 19°. Furthermore, Figure 6b shows that as the polymer content increased from 3.5% to 5.5%, the large-displacement interface adhesion force increased from 11 kPa to 19 kPa, while the corresponding friction angle decreased from 14° to 9°. Although no linear envelope was formed when the polymer content was 0%, the trends observed in Figure 6b for polymer content increasing from 3.5% to 5.5% are consistent with those shown in Figure 6a. However, it should be noted that, due to the limited number of normal stress levels and the absence of uncertainty bounds, the fitted *R*^2^ values and the derived apparent adhesion and friction angle should be interpreted with caution and regarded as descriptive parameters within the tested range rather than intrinsic material properties.

### 3.3. Vertical Deformation of GMTC/GCL or BPC-GCL Interfaces

**Figure 7:**
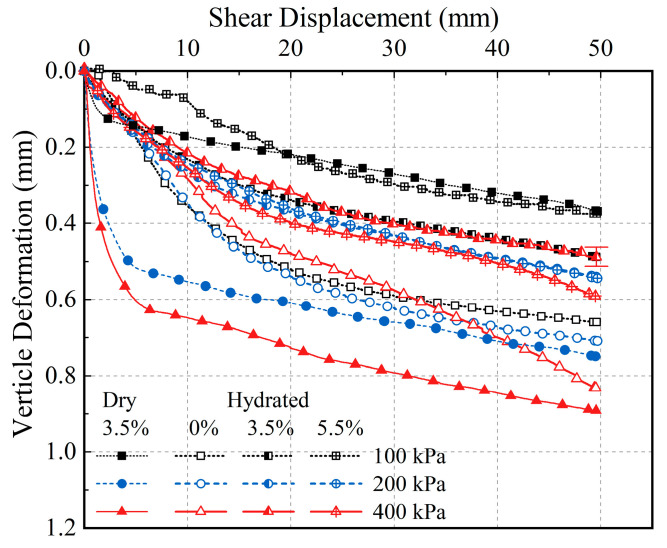
Vertical deformation-shear displacement curve of the GMTC/BPC-GCL interfaces with varying polymer content under dry and hydrated conditions.

Vertical deformation is practically important because it reflects interface compressibility and stability, and its analysis is essential for assessing serviceability, identifying potential failure mechanisms, and ensuring safe and reliable design of GCL-based barrier systems [2,6,12,13,19]. The relationship between vertical deformation and shear displacement for the GMTC/BPC-GCL interfaces with varying polymer content under dry and hydrated conditions at 100 kPa, 200 kPa and 400 kPa is shown in Figure 7, where compression is taken as a positive value. All GMTC/BPC-GCL interfaces exhibited monotonic compression during shearing, with vertical deformations remaining below 0.9 mm in all cases. Under dry conditions, the GMTC/BPC-GCL interfaces exhibited primary compression within the first 5 mm of shear displacement, followed by moderate compression of 0.20–0.30 mm thereafter. Under hydrated conditions, primary compression generally occurred within the first 15 mm of displacement, transitioning to moderate compression of 0.20–0.25 mm between 15 and 50 mm, except for the conventional GCL (0% polymer) at 400 kPa, where the curve did not stabilize even at 50 mm of shear displacement.

The vertical displacements of the BPC-GCL interface with 3.5% polymer content were compared under dry and hydrated conditions. At 100 kPa, the vertical displacement of the BPC-GCL interface was lower under dry conditions than under hydrated conditions, whereas at 200 and 400 kPa, the vertical displacement was greater under dry conditions. A comparison under hydrated conditions for varying polymer content showed that the conventional GCL (0% polymer) exhibited substantially larger vertical displacements than BPC-GCLs with 3.5% and 5.5% polymer. For BPC-GCLs, at 100 kPa, the vertical displacement was larger for 3.5% polymer than for 5.5% polymer. At 200 kPa, the vertical displacement at 3.5% was slightly higher than at 5.5% before 25 mm of shear, after which the values became comparable. At 400 kPa, the vertical displacement at 3.5% polymer content was smaller than at 5.5%, and with increasing normal stress, the vertical displacement at 3.5% gradually became less than that at 5.5%.

## 4. Mechanisms Contributing to Shear Behavior

**Figure 8:**
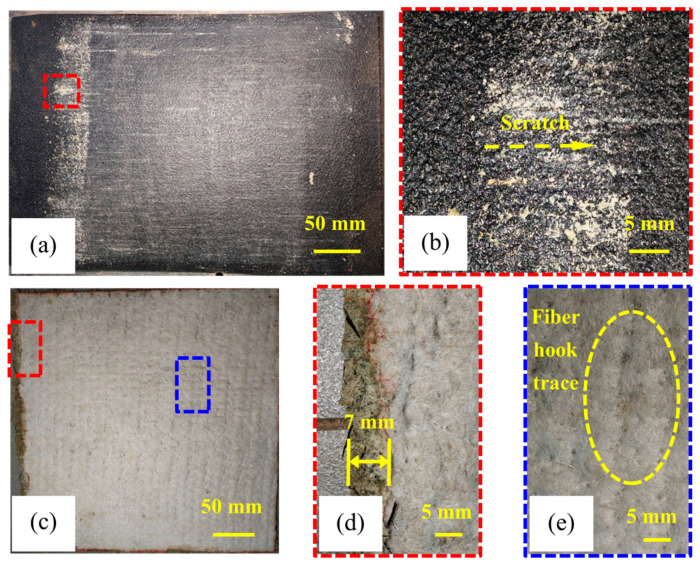
Photographs of the GMTC/BPC-GCL (3.5% polymer content) interface after shearing under dry conditions at 400 kPa: (**a**) GMTC surface, (**b**) close-up of scratches on the GMTC surface, (**c**) BPC-GCL surface, (**d**) close-up of BPC-GCL layers, (**e**) close-up of fiber hook traces on the BPC-GCL surface.

Figure 8 presents photographs of the GMTC/BPC-GCL (3.5% polymer content) interface after shearing under dry conditions at 400 kPa. As shown in Figure 8a, under dry conditions and high normal stress, a small amount of bentonite was extruded from the BPC-GCL and adhered to the GMTC surface during shearing. Additionally, numerous scratches from the BPC-GCL non-woven geotextile are visible on the GMTC (Figure 8b). As shown in Figure 8c, the BPC-GCL macroscopically exhibited no obvious overall deformation under dry conditions. According to Figure 8d, only a minor misalignment of approximately 7 mm occurred between the non-woven and woven geotextiles of the BPC-GCL under dry conditions. As shown in Figure 8e, the marks on the non-woven geotextile surface of the BPC-GCL, caused by the hooking and pulling of fibers against the GMTC texturing, were minimal under dry conditions. This indicates that no significant internal shear deformation occurred within the BPC-GCL during interfacial shearing, and the contribution of needle-punched fibers to force transfer was limited. The shear behavior under dry conditions was primarily governed by interfacial friction between the materials.

**Figure 9:**
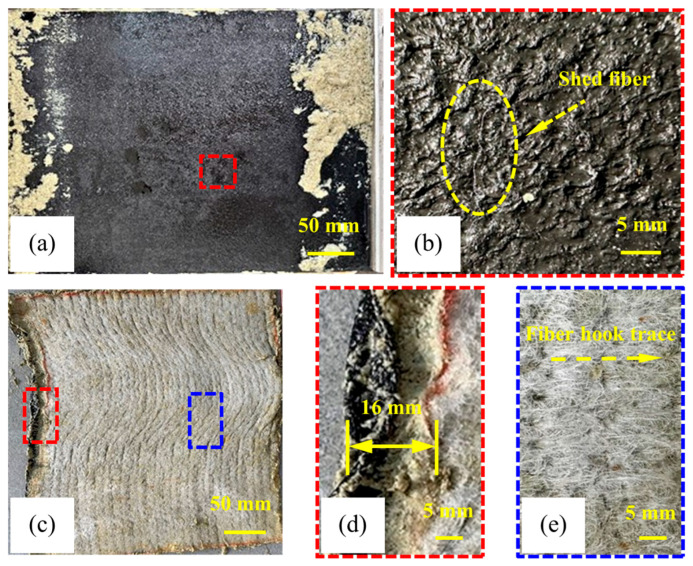
Photographs of the GMTC/BPC-GCL (3.5% polymer content) interface after shearing under hydrated conditions at 400 kPa: (**a**) GMTC surface, (**b**) close-up of shed fiber on the GMTC surface, (**c**) BPC-GCL surface, (**d**) close-up of BPC-GCL layers, (**e**) close-up of fiber hook traces on the BPC-GCL surface.

Figure 9 presents photographs of the GMTC/BPC-GCL (3.5% polymer content) interface after shearing under hydrated conditions at 400 kPa. Figure 9a reveals that under hydrated conditions, the amount of bentonite extruded from the BPC-GCL was substantially greater than under dry conditions. It can also be observed that fibers from the non-woven geotextile detached and adhered to the GMTC during shearing (Figure 9b). Additionally, it is evident from Figure 9c that the BPC-GCL underwent significant overall deformation and structural shifting under hydrated conditions. As shown in Figure 9d, a considerable misalignment of about 16 mm occurred between the woven and non-woven geotextile sides of the BPC-GCL under hydrated conditions at 400 kPa normal stress. These observations indicate that significant internal damage occurred within the GCL during interfacial shearing under hydrated conditions. A large number of needle-punched fibers fractured, resulting in a loss of mechanical interlock for the bentonite. As a consequence, bentonite was retained at one end of the GMTC and extruded from the opposite end under normal stress. Furthermore, under hydrated conditions, the marks on the BPC-GCL non-woven geotextile surface caused by the hooking and pulling of fibers against the GMTC surface filaments were more pronounced than under dry conditions (Figure 9e), indicating that interfacial shear failure also occurred in the hydrated case.

The distinct shear behaviors observed under dry and hydrated conditions stem from hydration-induced changes in bentonite properties and structural interactions. Under dry conditions, the BPC-GCL exhibits higher stiffness, maintaining a relatively intact macroscopic structure with minor misalignment, which promotes stronger mechanical interlocking and interfacial friction. In contrast, hydration softens the BPC-GCL and facilitates polymer migration to form a lubricating layer [18,22,23,24,25]. This softening and lubrication reduce frictional resistance and facilitate interlayer sliding, leading to the severe 16 mm internal misalignment, substantial bentonite extrusion, and pronounced fiber pull-out (Figure 9). Ultimately, under hydrated conditions, the shear strength transitions to being primarily controlled by internal shear damage. The manifestation of these mechanisms is highly dependent on the applied normal stress. At low stress levels (100 kPa), the softened hydrated bentonite dominates volumetric changes and shear behavior. The hydrated bentonite yields higher large-displacement shear strength (see Figure 4), which is related to the inherent adhesive properties of the hydrated matrix [6,9,11,15]. During shearing, this softened bentonite undergoes sustained lateral flow, increasing compressive deformation. Conversely, compression in the dry BPC-GCL at low stress is largely confined to the initial shearing stage (within approximately 5 mm of displacement). The low stress is insufficient to induce substantial compression, resulting in a smaller overall vertical displacement (see Figure 7). As normal stress increases, the behavior diverges significantly. Under dry conditions, high stress enhances the mechanical interlocking and friction between the GMTC texture and the nonwoven geotextile. To overcome this strong interfacial engagement, continuous vertical compression is required to accommodate horizontal displacement, leading to larger vertical displacements (see Figure 7). Under hydrated conditions, however, the bentonite softening and interfacial lubrication weaken the interlocking mechanisms and exacerbate strength reduction (see Figure 4) [6,9,11,15,18,22,23,24,25]. Simultaneously, the softened hydrated bentonite tends to flow laterally (extrusion) rather than compress vertically, as clearly observed in the post-shear photographs (Figure 9). Furthermore, significant hydration swelling and initial compression during the preloading and 24 h consolidation period (Section 2.3) substantially reduce the available void space, thereby limiting additional vertical deformation during shearing (see Figure 7).

**Figure 10:**
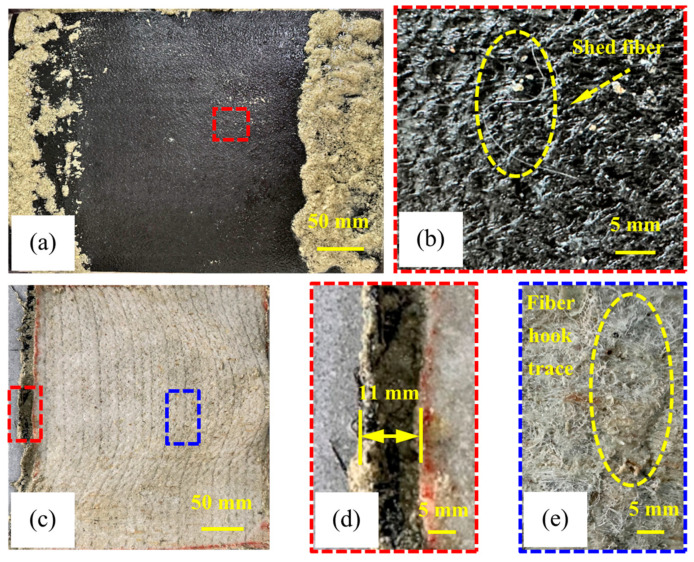
Photographs of the GMTC/BPC-GCL (5.5% polymer content) interface after shearing under hydrated conditions at 400 kPa: (**a**) GMTC surface, (**b**) close-up of shed fiber on the GMTC surface, (**c**) BPC-GCL surface, (**d**) close-up of BPC-GCL layers, (**e**) close-up of fiber hook traces on the BPC-GCL surface.

**Figure 11:**
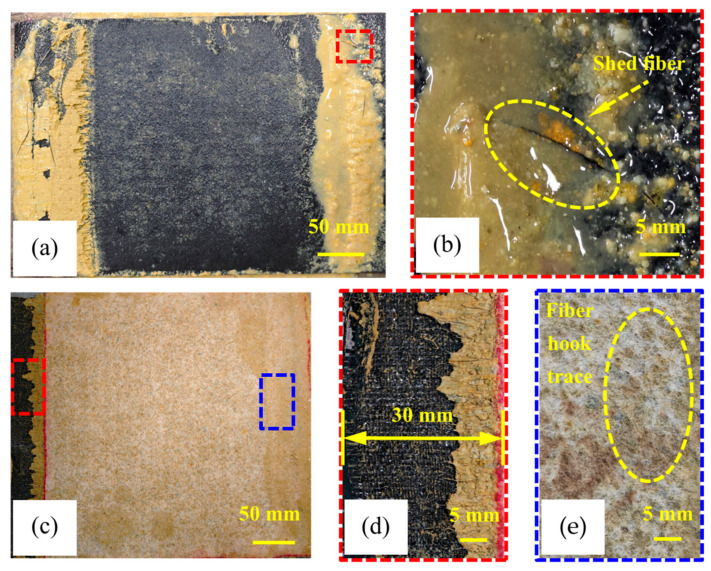
Photographs of the GMTC/ GCL interface after shearing under hydrated conditions at 400 kPa: (**a**) GMTC surface, (**b**) close-up of shed fiber on the GMTC surface, (**c**) GCL surface, (**d**) close-up of GCL layers, (**e**) close-up of fiber hook traces on the GCL surface.

Figure 10 presents photographs of the GMTC/BPC-GCL (5.5% polymer content) interface after shearing under hydrated conditions at 400 kPa. As shown in Figure 10a, the BPC-GCL with 5.5% polymer content showed a moderate amount of extruded bentonite, which was slightly greater than that of the 3.5% BPC-GCL in Figure 9, and was accompanied by fiber detachment from the non-woven geotextile adhering to the GMTC (Figure 10b). Furthermore, Figure 10c illustrates that the BPC-GCL with 5.5% polymer content experienced a minor overall deformation. In comparison, the BPC-GCL with 5.5% polymer content displayed a misalignment of about 10 mm between the woven and non-woven geotextile sides (Figure 10d), which is slightly smaller than the 16 mm observed for the 3.5% BPC-GCL in Figure 9. A comparison of subfigures (e) in Figure 9, Figure 10 and Figure 11 reveals no noticeable hooking or pulling marks on the non-woven surface of the conventional GCL, whereas such marks were clearly visible on both the 3.5% and 5.5% BPC-GCLs—most pronounced in the 3.5% specimen. This suggests that internal damage in the polymer-modified BPC-GCLs was accompanied by interfacial shear failure.

Figure 11 presents photographs of the GMTC/GCL interface after shearing under hydrated conditions at 400 kPa. As shown in Figure 11a, the conventional GCL (0% polymer) exhibited the largest amount of extruded bentonite among the three compositions under hydrated high normal stress, along with detachment of the woven geotextile from the GCL onto the GMTC (Figure 11b). As shown in Figure 11c, the conventional GCL (0% polymer) macroscopically exhibited no obvious overall deformation; however, bentonite was clearly observed extruding through the voids of the non-woven geotextile surface. According to Figure 11d, the conventional GCL experienced the largest misalignment, approximately 30 mm, between its non-woven and woven geotextiles under high normal stress, indicating severe internal damage.

The shear behavior of GMTC/BPC-GCL interfaces under varying polymer content is strongly influenced by polymer-induced changes in bentonite hydration and macroscopically observed structural damage. At low (100 kPa) and intermediate (200 kPa) normal stresses, polymer content has little effect, with shear strength remaining similar across compositions. At 400 kPa, peak shear strength shows a non-linear trend, initially increasing and then decreasing with polymer content (Figure 4). In BPC-GCL, the incorporation of polymer limits massive internal disruption, eliminating the initial weak peak observed in conventional GCL and enabling shear resistance to develop continuously. Moderate polymer content (3.5%) retains internal structure and interfacial engagement, shifting failure to a combined internal and interfacial mode characterized by internal deformation (16 mm misalignment) and sustained interfacial friction (Figure 9e), improving compressive performance, and producing the highest peak shear strength. The lower performance of the 5.5% BPC-GCL may be attributed to over-modification effects and the fact that the polymer hydrogel may be more readily squeezed out during shearing, which further promotes interface slippage (Figure 10a) [18,22,23,24,25]. However, this conclusion lacks direct supporting evidence, which represents a limitation of the present study and requires further in-depth investigation. For conventional GCL (0% polymer), hydration under high stress leads to catastrophic internal structural failure rather than interfacial friction, evidenced by the massive 30 mm internal misalignment and the almost absence of fiber hooking traces (Figure 11), followed by rapid yielding, extensive lateral bentonite extrusion, and extensive plastic deformation, resulting in the lowest peak shear strength and largest vertical displacement (Figure 4 and Figure 7) [18,22,23,24].

## 5. Summary and Conclusions

A comprehensive experimental study was conducted to evaluate the interface shear behavior between a GMTC and both conventional GCL and BPC-GCL under dry and hydrated conditions, with polymer contents of 0%, 3.5%, and 5.5%.

The conclusions are as follows:

(1) Hydration significantly reduces both peak and large-displacement interface shear strengths, particularly under high normal stress. Polymer content non-linearly modulates the shear response under hydrated conditions. The 3.5% BPC-GCL exhibited highest shear strength under the tested conditions, yielding a 18% higher peak strength compared to the conventional GCL (0% polymer) at 400 kPa, whereas the 5.5% composition leads to an 9% strength reduction relative to the 3.5% polymer content.
(2) The large-displacement to peak strength ratio is generally lower under dry conditions, indicating pronounced post-peak strength loss. Hydration increases this ratio, reflecting a transition to more ductile internal deformation. At a high normal stress of 400 kPa, the 3.5% BPC-GCL maintains the highest LD/peak ratio with superior retained post-peak capacity. The 5.5% BPC-GCL exhibits an intermediate LD/peak ratio, while the conventional GCL suffers a sharp decrease corresponding to the macroscopically observed severe internal structural disruption.
(3) Interface strength envelopes are predominantly linear under the tested stress range. Hydration significantly reduces the apparent interface friction angle while increasing the apparent adhesion. Under hydration, as the polymer content increases from the conventional GCL to the 3.5% BPC-GCL and further to the 5.5% BPC-GCL, the apparent adhesion initially decreases and subsequently increases, while the apparent friction angle initially increases and subsequently decreases.
(4) Vertical deformation during shear is primarily compressive. Dry interfaces compress predominantly during the initial shearing stage (<5 mm). Under hydrated conditions and high normal stress, the conventional GCL exhibits the largest vertical displacement, which corresponds to the extensive lateral bentonite extrusion observed during shearing. In contrast, polymer-modified BPC-GCL, especially at 3.5% polymer content, demonstrates restrained compression, consistent with the macroscopically observed reduction in volumetric change and internal damage.
(5) The underlying shear failure mechanisms shift distinctly with hydration conditions and polymer content. Dry interfaces fail through mechanical interlocking and interfacial friction. Hydration softens the bentonite, introducing lubrication and shifting the failure toward internal BPC-GCL damage. The 3.5% polymer content facilitates a composite failure mode that effectively combines internal structural resistance with sustained interfacial friction. Conversely, the conventional GCL fails through severe internal damage; while the 5.5% polymer content also exhibits a combined internal and interfacial failure mode, it demonstrates a reduction in shear resistance driven by excessive interfacial lubrication.

## Figures and Tables

**Table 1:** The physical properties of the GCL.

GCL Type	Thickness (mm)	Bentonite Mass/Area (g/m^2^)	Non-Woven Geotextile (g/m^2^)	Woven Geotextile (g/m^2^)	Peel Strength (N/m)	Polymer Content (%)
GCL	7	4800	200	110	650	0
3.5% BPC-GCL	7	6000	200	110	790	3.5
5.5% BPC-GCL	7	6000	200	110	790	5.5

**Table 2:** The physical properties of the HDPE geomembranes.

GM Type	Thickness (mm)	Density (kg/m^3^)	Tensile Strength at Yield (N/mm)	Tensile Elongation at Yield-Break (%)	Asperity Height (mm)	Shore Hardness (HA)
GMTC	2	950	29	13	0.25	93.6

**Table 3:** Test condition.

Shear Interface	Dry and Hydrated Conditions	Polymer Content (%)	Normal Stress (kPa)
GMTC/BPC-GCL	Dry	3.5	100 200 400
	Hydrated	0 3.5 5.5	

## Data Availability

The raw data supporting the conclusions of this article will be made available by the authors on request.
